# Cannabidiol’s Multifactorial Mechanisms Has Therapeutic Potential for Aneurysmal Subarachnoid Hemorrhage: a Review

**DOI:** 10.1007/s12975-022-01080-x

**Published:** 2022-09-15

**Authors:** Nicholas Henry, Justin F. Fraser, Joseph Chappell, Tamra Langley, Jill M. Roberts

**Affiliations:** 1 College of Medicine, University of Kentucky, Lexington, KY USA; 2 Department of Neurosurgery, University of Kentucky, Lexington, KY USA; 3 Department of Neuroscience, University of Kentucky, Lexington, KY USA; 4 Department of Radiology, University of Kentucky, Lexington, KY USA; 5 Department of Anesthesiology, University of Kentucky, Lexington, KY USA; 6 Department of Neurology, University of Kentucky, Lexington, KY USA; 7 Center for Advanced Translational Stroke Science, University of Kentucky, Lexington, KY USA; 8 College of Pharmacy, Department of Pharmaceutical Sciences, University of Kentucky, Lexington, KY USA

**Keywords:** Stroke, Inflammation, Vasospasm, CBD

## Abstract

Subarachnoid hemorrhage (SAH) is a major health burden that accounts for approximately 5% of all strokes. The most common cause of a non-traumatic SAH is the rupture of a cerebral aneurysm. The most common symptom associated with SAH is a headache, often described as “the worst headache of my life.” Delayed cerebral ischemia (DCI) is a major factor associated with patient mortality following SAH and is often associated with SAH-induced cerebral vasospasm (CV). Cannabidiol (CBD) is emerging as a potential drug for many therapeutic purposes, including epilepsy, anxiety, and pain relief. We aim to review the potential use of CBD as a treatment option for post-SAH critically ill patients. Through a literature review, we evaluated the known pharmacology and physiological effects of CBD and correlated those with the pathophysiological outcomes associated with cerebral vasospasm following subarachnoid hemorrhage. Although overlap exists, data were formatted into three major categories: anti-inflammatory, vascular, and neuroprotective effects. Based on the amount of information known about the actions of CBD, we hypothesize the anti-inflammatory effects are likely to be the most promising therapeutic mechanism. However, its cardiovascular effects through calcium regulation and its neuroprotective effects against cell death, excitotoxicity, and oxidative stress are all plausible mechanisms by which post-SAH critically ill patients may benefit from both early and late intervention with CBD. More research is needed to better understand if and how CBD might affect neurological and vascular functions in the brain following injury such as subarachnoid hemorrhage.

**Supplementary Information:**

The online version contains supplementary material available at 10.1007/s12975-022-01080-x.

## Introduction

The most common cause of a non-traumatic subarachnoid hemorrhage (SAH) is the rupture of a cerebral aneurysm [1]. The fatality rate of SAH patients within the first 28 days can be as high as 42%, while 10–20% of patients die before reaching the hospital [2, 3]. In aneurysmal subarachnoid hemorrhage (aSAH), the cardinal symptom is a severe, sudden headache [4], and the most common risk factors are hypertension, smoking, and extensive alcohol consumption [5]. Females in specific populations and first-degree relatives with SAH are also associated risk factors [6, 7]. Patients that survive the initial bleed have outcomes that range from minor cognitive deficits to severe neurological disability.

Delayed cerebral ischemia (DCI) is a potentially fatal condition that occurs in the subacute phase of SAH episodes. DCI affects approximately 30% of patients and is the leading cause of morbidity and mortality after surviving the initial aneurysm rupture [8]. Characterized by an acute reduction of arterial blood supply to the brain, DCI is dependent on the severity of the initial aneurysm, early brain pathologies, and the development of cerebral vasospasm (CV), though other factors such as microvascular spasm and micro-thrombosis have been considered contributary [9, 10]. CV, the constriction of intracranial arteries that usually begins 3 days after SAH and can last up to 2–3 weeks, was once considered the singular cause of DCI, though this has since been challenged after incongruencies between the presence of CV and evidence of DCI were observed [11, 12]. It is, however, still identified as a major contributor to DCI. An increase in intracellular calcium is thought to play an important role in CV due to its characteristic vasoconstriction effects in smooth-muscle cells; however, many other conducive mechanisms have been described, including the nitric oxide pathway, endothelin-induced vasoconstriction, pro-inflammatory cascades, hypoxia-inducible factor-1 (HIF-1) transcriptional modulation, oxidative stress, and apoptosis from early brain injury (EBI), among others [13–15]. Nimodipine, a calcium channel blocker, is one of the few current standard therapies following SAH as it has shown efficacy in reducing neurologic deficits from DCI, an effect believed to be due to the prevention of CV, though evidence of this mechanism via angiography visualization has been variable [16]. Other treatments consist of blood pressure maintenance, including induced hypertension when the CV is present, and neurovascular intervention (intra-arterial administration of spasmolytics and balloon angioplasty) if needed [17, 18]. Pain management includes acetaminophen/caffeine/butalbital cocktail and opioids [19]. Despite these treatment protocols, poor patient outcomes persist. Given there are many pathological mechanisms at play following SAH that significantly impact morbidity and mortality, research has focused on determining which pathways may have a key role in the pathophysiology behind CV and DCI in order to evaluate new treatment options.

Cannabidiol (CBD) is the dominant phytocannabinoid that accumulates in hemp, a plant closely related to marijuana that contains a much lower amount (< 0.3% dry weight) of the psychoactive cannabinoid tetrahydrocannabinol (THC). Unlike THC, CBD does not cause euphoria or intoxication, making it an attractive drug for daily therapeutic use [20]. First used for the treatment of pain, preclinical reports now demonstrate tissue-protective and anti-inflammatory effects in models of colitis, kidney injury, cardiovascular disease, arthritis, and cancer [20–24]. It has also been FDA-approved for the treatment of specific types of pediatric epilepsy after clinical trials found it to be more effective than conventional agents as well as therapeutically additive when used as an adjunctive agent [25–27]. The most common side effects include diarrhea, weight loss, transaminase elevations, and sleep disturbance, while showing little evidence of any severe adverse side effects [28]. However, many of CBD’s pharmacologic mechanisms and targets are still undetermined, so further research into its long-term side effects and therapeutic potential needs evaluation before definitive conclusions are drawn. This literature review will evaluate the potential use of CBD as a treatment option for post-SAH critically ill patients based on the correlations between the known pharmacology and physiological effects of CBD and the pathologies associated with SAH, most notably CV and DCI.

## Methods

A PubMed database search for English-language papers published up to July 2022 was conducted using the following search terms: “cannabidiol” or “CBD” and “subarachnoid hemorrhage” or “vasospasm” or “cerebral vasospasm” or “mechanism” or “physiology” or “vascular” or “pathway” or “inflammation” or “brain”. Selection criteria were used for all CBD-related articles that support the findings of this review, which includes studies published since the year 1998 with original experimental data from preclinical or clinical models that examine the physiologic or molecular mechanisms of CBD. Due to the rather low number of articles describing CBD’s effects on the brain, and more specifically hemorrhagic disease, all published articles that abide by the above criteria and provide pertinent information were used, despite the anatomical feature or pathology being explored, and the limitations associated with these comparisons were addressed. The following referenced review articles were found using the search terms described above and used to identify supplemental articles that further describe CBD- and SAH-related mechanisms [15, 29–35]. Any review articles that are referenced in the results section are non-contributary to the results of this review and therefore solely act to introduce topics or provide pertinent supplemental knowledge.

## Results: CBD Targets Relevant to SAH

CBD has over 65 molecular targets with varying effects on each of those molecules [30]. While some animal studies have shown conflicting results when compared to studies with humans, there are multitudes of CBD targets that are relevant to SAH pathologies. Although crossover exists, we have grouped these targets into three major classifications: anti-inflammatory, vascular, and neuroprotective. This review will assess evidence from preclinical and clinical studies pertaining to the hypothesized therapeutic potential of CBD following SAH. To date, no studies that specifically look at CBD’s use in SAH were found, so the following results are drawn from CBD’s use in other disease models and compared to established mechanisms in SAH pathophysiology.

### Anti-inflammatory Effects

Following the rupture of an aneurysm and leakage of blood into the subarachnoid space, a number of events take place that cause both local and systemic inflammation. As red blood cells degrade, free hemoglobin stimulates the upregulation of specific cell adhesion molecules on the luminal surface of endothelial cells [36]. This allows macrophages and neutrophils to bind to endothelial cells through rolling adhesion and enter the subarachnoid space, where they phagocytose extravasated hemoglobin-haptoglobin complexes [37]. However, macrophages/neutrophils remain trapped in the subarachnoid space, and as they begin to die and degranulate, a multitude of inflammatory factors are released, including free radicals, cytokines, and endothelins, leading to arterial vasoconstriction [14, 37, 38]. In addition, microglial cell activation near the site of hemorrhage upregulates Toll-like receptors, which leads to an increase in high-mobility group box-1 (HMGB1) protein and downstream activation of nuclear factor kappa beta (NF-κβ) [34, 39, 40]. This, in turn, causes the release of pro-inflammatory cytokines such as interleukin-1 beta (IL-1β), IL-6, and tumor necrosis factor alpha (TNF-α), all of which are found to be elevated in both the cerebrospinal fluid (CSF) and serum of SAH patients [34]. These inflammatory processes also lead to increased levels of matrix metalloproteinase 9 (MMP-9), which degrades the extracellular matrix surrounding blood vessels and leads to blood-brain-barrier (BBB) disruption and leakage of inflammatory mediators into the brain parenchyma [41]. CBD produces anti-inflammatory effects in numerous in vivo and in vitro studies, primarily resulting from inhibiting the activity of molecules involved in the regulation of the inflammatory response and prevention of leukocyte proliferation. A summary of the studies depicting CBD’s anti-inflammatory effects is provided in Online Reference 1 (Table OR1). The following subsections describe how CBD might modulate the production and activity of these molecules.

### Microglia

Microglia are parenchymal macrophages responsible for the tight regulation of the brain’s microenvironment. These immune cells are primarily responsible for the phagocytosis of cells and other debris, a function that may be beneficial in clearing blood products following SAH. Hassan et al. [42] show that CBD treatment of microglial cells leads to increased phagocytosis, at least in part, via activation of transient receptor potential cation channel subfamily V (TRPV) receptor channels and modulation of intracellular calcium influx. Following the release of pro-inflammatory cytokines in SAH, activated microglia may polarize from a homeostatic state into pro-inflammatory (with surface markers CD16, CD86, iNOS) or anti-inflammatory (with surface markers CD206, CD163, Arg1, MHCII) phenotypes that modulate local inflammation through the release of pro-inflammatory (IL-6, TNF-α) and anti-inflammatory (IL-10) mediators, respectively [43]. This polarization process, accompanied by morphologic changes of the microglia themselves, has been observed to dynamically shift in SAH, where pro-inflammatory phenotypes accumulate in the brain parenchyma shortly after hemorrhage (days 1–3) with subsequent transition to anti-inflammatory phenotypes as pathology progresses (days 5–10) [44]. Recent evidence supports a pathologic role of pro-inflammatory microglia on brain damage after SAH, and studies aiming to combat this neuroinflammation and increase anti-inflammatory microglial expression in SAH models have shown improved outcomes [45, 46]. As such, a reduction in the pro-inflammatory microglial processes may be of benefit.

Studies show that CBD treatment inhibits LPS-induced microglial inflammation in vitro by reducing the production and release of pro-inflammatory molecules (e.g., IL-1β, IL-6, TNF-α, and IFN-β), as well as upregulating genes (e.g., *Trib3, Dusp1*) that downregulate pro-inflammatory transcription factors such as NF-κβ [47–51]. Dos-Santos-Pereira et al. [51] further identified the mechanism behind these in vitro effects as being predominantly receptor-independent and mediated by inhibition of ROS production and the NF-κβ signaling pathway, as well as attenuating LPS-induced increases in glucose consumption. As no studies were found to explicitly explore microglial polarization, CBD’s effects on many of the microglial differentiating surface markers were not identified. It has, however, been shown that CBD can modulate microglial activity. In rodent models of viral-induced multiple sclerosis, Alzheimer’s disease, and cerebral ischemia, CBD administration reduced microglial activation [52–54]. Interactions with cannabinoid and adenosine A2A receptors as well as modulation of intracellular calcium were suggested mechanisms in the Alzheimer’s model, though this may vary between pathologies [54]. Carrier et al. [55] identified CBD as a competitive inhibitor of adenosine, further suggesting CBD’s immunosuppressive actions in microglia are mediated by the enhancement of endogenous adenosine signaling. Other studies have demonstrated that treatment of CBD in BV-2 microglial cells extensively alters gene expression (680 upregulated genes and 524 downregulated genes were described), while it was suggested that the immunosuppressive and anti-neoplastic effects of CBD occur by inhibiting the conductance of mitochondrial voltage-gated anion channel 1 (VDAC1), inducing altered BV-2 cell mitochondrial function and cell death [56, 57].

### Adhesion Molecules

As the local inflammatory response follows SAH, the concentration of various adhesion molecules important for mediating phagocyte migration and infiltration increases, a process pivotal in propagating neuroinflammation. Vascular adhesion molecule 1 (VCAM-1) is an adhesion molecule that is upregulated by inflammatory cytokines (TNF-α, IL-1) and mediates leukocyte adhesion to vascular endothelium. Human studies have identified increased serum and CSF levels days following SAH, and though an association between serum VCAM-1 levels and CV, DCI, or 3-month patient outcomes was not found, leukocyte adhesion remains a significant contributing factor towards post-SAH neuroinflammation [58, 59]. Mecha et al. [53] showed that CBD decreased the transmigration of leukocytes in a viral model of multiple sclerosis, partially by downregulating VCAM-1. They found that administration at the time of infection produced the best anti-inflammatory results, and that the adenosine A2A receptor is partially involved in this process. CBD also decreased VCAM-1 levels in human brain microvascular endothelial cells exposed to oxygen–glucose deprivation (OGD) in an in vitro model of BBB [60]. This effect was inhibited by a peroxisome proliferator-activated receptor gamma (PPARγ) antagonist and reduced by a serotonin 1-A receptor (5-HT_1A_) antagonist, suggesting CBD’s effect on VCAM-1 includes a mechanism mediated by the activation of these two receptors [60]. While there is evidence for CBD modulating 5-HT1A and PPARγ activities [30, 60–63], direct CBD binding has yet been reported. Molecular docking simulations of CBD binding to these receptors have been suggestive of direct interactions (data not shown), but remain to be experimentally substantiated [64]. Reduction of VCAM-1 and intercellular adhesion molecule 1 (ICAM-1) by CBD treatment has also been reported in an animal model of diabetes-induced cardiomyopathy, high glucose-induced endothelial cell barrier disruption, and in TNF-α exposed sinusoidal epithelial cell assays [65–67].

### Cytokines

#### Interleukins

Interleukins are cytokines that are particularly important for modulating the immune response by acting on and being released by various immune cells. There is significant evidence supporting interleukin release (i.e., IL-1, IL-6, IL-8) as critical mediators of the inflammatory response following SAH [34]. In fact, serum concentrations of IL-1Rα, the alpha subunit of the IL-1 receptor, were significantly associated with 1-year poor outcome [68]. Mecha et al. [53] evaluated the effects of CBD in an astrocyte culture and reported anti-inflammatory effects partially due to the downregulation of IL-1β and TNF-α gene expression. When added to microglial BV-2 cells treated with LPS, CBD treatment decreased the production and release of IL-1β and IL-6 [48]. There is also evidence of similar anti-inflammatory effects of CBD in vivo, as levels of interleukins were reduced following CBD treatment in a model of cisplatin-induced nephropathic inflammation and hypoxia–ischemia [32, 69]. Pre- and post-treatment of CBD in a rat TBI model significantly reduced IL-1β levels and mitigated BBB disruption [70]. Juknat et al. [47] revealed in their LPS-stimulated gene modification experiment that CBD reduces LPS-induced upregulation of IL-1β, IL-1α, and IL-27 by 81%, 68%, and 62%, respectively. Interestingly, the production of the anti-inflammatory cytokine, IL-10, was also decreased by CBD administration in both in vitro and in vivo experiments [71]. While the mechanism of action remains unclear, CBD may act through various receptor types, such as cannabinoid receptor type 2 (CB_2_), 5-HT_1A_ receptors, and adenosine A2A receptors [72, 73]. Despite findings demonstrating a role for CB_2_, it has been found that CBD has a low affinity for CB_2_ and may even act as an antagonist to CB_2_ [74, 75]. Additionally, CB_2_ expression is most prominent in B cells and natural killer cells located in the immune system (e.g., spleen), while it has only limited expression in the brain, indicating a need for more assessment on the potential role of CB_2_ in mediating CBD’s effects on interleukins [76].

#### TNF-α

TNF-α is an acute phase pro-inflammatory cytokine that is believed to play a role in the development of cerebral vasospasm following SAH. Increased levels of TNF-α are present in the serum and CSF of SAH patients, and early elevations of serum levels have even been associated with worse outcomes [34, 77]. Both in vitro and in vivo studies have demonstrated CBD’s ability to decrease TNF-α concentrations [53, 69]. In LPS-treated retinal BV-2 cells and in ex vivo inflammatory models, CBD-inhibited TNF-α production is thought to be dependent on the adenosine A2A receptor [49, 55]. Malfait et al. [24] found that both CBD ex vivo administration in knee synovial cells and in vivo administration in mice reduced TNF-α release and LPS-induced levels, respectively. CBD also attenuated TNF-α production in isolated Kupffer cells and reduced apoptotic damage in hypoxia–ischemia, in part by downregulating TNF-α expression in the brain and CSF [65, 73, 78]. Finally, studies have found that CBD administration at 1 h, 25 h, and 49 h following ischemia/reperfusion and 30 min before and 6 h after induced TBI reduced serum and brain TNF-α levels, respectively [70, 79].

#### High-Mobility Box Group 1

High-mobility box group 1 (HMBG1) is a protein secreted by immune cells to mediate cytokine release by phagocytes, in particular microglia activation. HMBG1 levels are significantly increased in the CSF of SAH patients and are independently associated with poor outcomes and neuronal cell death [80]. Few studies have evaluated the effects of CBD on HMBG1, but those that have demonstrate similar results. In a mouse model of middle cerebral artery occlusion (MCAO), CBD treatment reduced neurological impairment following stroke by inhibiting myeloperoxidase (MPO) containing cell expression of HMBG1, inhibiting macrophage/monocyte expression of HMBG1, reducing HMBG1 plasma levels, and preventing glial activation [52]. A similar study found that CBD produced neuroprotection and reduced HMBG1 plasma levels in MCAO mice when administered 1 and 3 (but not 5) days post-stroke [81].

### Transcription Factors

NF-κβ is a protein complex important for modulating the immune response by regulating DNA transcription, cytokine production, and cell survival. It is also known to be significantly upregulated following SAH [34]. CBD treatment reduces NF-κβ activation in animal models of hepatic ischemia/reperfusion and diabetes [65–67, 79]. When LPS is applied to activate microglial BV-2 cells in vitro, CBD administration upregulates *Trib3*, a negative regulator of NF-κβ, thereby reducing the activity of the NF-κβ pathway [47, 48]. It was also shown that CBD reduces NF-κβ activation by partially reversing the LPS-induced degradations of IRAK-1 and the downstream signaling protein and NF-κβ inhibitor, Iκβ [48].

The Janus kinase (JAK) and signal transducer and activator of transcription (STAT) protein pathway is a cascade of interactions that transduce signals from the cell surface that modulate gene/protein expression in response to extracellular cytokine/interferon binding. It has been observed that therapeutic activation of the JAK1/STAT3 pathway was protective against EBI following SAH [82]. While there are few reports, studies do show CBD is able to regulate this pathway. Juknat et al. [47] report that CBD upregulated STAT3, a transcription regulator with anti-inflammatory roles in macrophage and neutrophil activity, in LPS-stimulated BV-2 cells [48]. They also found CBD decreased the activation of STAT1, a key molecule in the interferon-β (IFN-β) pro-inflammatory pathway [48]. Others found that CBD attenuated LPS-induced upregulation of STAT1 (− 25%), STAT2 (− 14%), Socs3 (− 59%), and Cish (− 59%), all of which are genes involved in the JAK/STAT pathway [47].

### Vascular Effects

Approximately half of SAH patients will experience CV in the days following aneurysm rupture, and this is one of the major reasons for the long in-hospital stay of all SAH patients. It is unclear why some patients develop vasospasm while others do not; evidence suggests the inflammatory response that occurs following SAH (as discussed above) may be a contributing factor. However, a number of other factors control vascular function, and CBD has been shown to effectively regulate some of these. A summary of the reviewed studies that demonstrated CBD’s vascular effects is provided in Online Reference 2 (Table OR2).

### Hemodynamics and Calcium

The release of reactive oxygen species and oxyhemoglobin following a bleed contributes to hemodynamic stress and vascular effects, such as CV, by altering the expression of calcium channels and increasing intracellular calcium [83–85]. Ishiguro et al. [86] showed oxyhemoglobin enhances small cerebral artery constriction and voltage-gated potassium channel (Kv) suppression acutely, while chronic exposure enhances the expression of the voltage-dependent calcium channels (VDCC). They suggest the acute and chronic effects of oxyhemoglobin act synergistically to alter channel activity and increase intracellular calcium levels. In a study using hippocampal cultures in high excitatory states, CBD reduces intracellular calcium and prevents calcium oscillations in a mitochondria-dependent manner [87]. CBD produced relaxation of the rat’s small mesenteric artery and retinal microvasculature in the setting of endothelin-1-induced vasoconstriction via calcium-dependent potassium (K_Ca_) and calcium channels [88, 89]. In addition, CBD produced vasorelaxation of the femoral artery in a rat model of diabetes by enhancement of cyclooxygenase (COX) activity, leading to the production of vasodilator prostanoids acting at the EP_4_ receptor [90]. Together, these data suggest CBD acts to restore calcium homeostasis and production of vasodilatory factors under pathological conditions, which indicates CBD may reduce the development of CV following SAH.

### Ischemia

As one of the devastating processes involved in SAH pathophysiology, ischemic damage often occurs both early after ictus as a result of increased intracranial pressure (ICP), decreased cerebral perfusion pressure (CPP), and decreased cerebral blood flow (CBF), as well as sub-acutely in the context of DCI. Contrasted to the abrupt cessation and restoration of CBF seen in ischemic strokes that can cause ischemia/reperfusion (I/R) injury, SAH has a more delayed ischemic and reperfusion process that has not been described to cause I/R injury. However, transient global cerebral ischemia, commonly caused by cardiac arrest and SAH, does result in similar pathophysiological changes in cerebral microcirculation, including vascular constriction, increased inflammation, BBB disruption, thrombus formation, and cell death [91]. Τhis distinction is of particular importance to this review since many of the referenced articles used ischemic stroke models, while no studies were found to explore SAH.

CBD displays the promising potential of protecting against ischemia. In MCAO mouse models, both pre- and post-ischemic administration of CBD was shown to reduce infarct size and improve neurologic scores, functional deficits, and survival rates [52, 81, 92, 93]. A therapeutic window of within 3 days post-insult was described in one study [81]. Intravenous administration of CBD before reperfusion protects against acute myocardial infarction in rabbits following 90-min coronary artery occlusion/24-h reperfusion [94]. CBD also produced cardioprotective effects in rats subjected to myocardial ischemia/reperfusion by reducing ventricular arrhythmias and attenuating reperfusion-induced infarction [95]. Interestingly, a separate study confirmed those results and suggested that CBD actions are mediated by the activation of the adenosine A1 receptor, since antagonizing this receptor inhibited the CBD response [96]. Since patients are at increased risk of both thrombotic and hemorrhagic events following SAH, specific considerations are made when deciding which therapeutic agents are used. Of importance, CBD was not associated with induction of thrombosis or platelet activation when studied in isolated platelets in vitro [97].

### Neuroprotective Effects

The term “early brain injury” (EBI) has been used to describe the mechanisms of acute neurologic deterioration after SAH, which includes cell death, cerebral edema, and neuronal dysfunction [98]. These mechanisms can lead to long-term complications such as memory impairment, epilepsy, neuropsychiatric disturbances, neurocognitive dysfunction, and focal deficits [99]. The mechanisms that lead to EBI remain unclear, but increased neuronal dysfunction and death are observed throughout the brain following SAH. Identification of a therapeutic that reduces cell death would provide great benefit to patient outcome and long-term quality of life. A summary of the neuroprotective effects of CBD is provided in Online Reference 3 (Table OR3).

### Excitotoxicity

Excitotoxicity, considered the main toxic mechanism in hypoxic-ischemic (HI) brain injury, occurs when metabotropic and ionotropic glutamate receptors are excessively activated with associated intracellular calcium influx via overstimulation of N-methyl-D-aspartate (NMDA) glutamate transporters [100], leading to neurotoxicity and cell death. Excitotoxicity can occur within minutes to hours following SAH, and studies have associated glutamate concentrations in the CSF with the development of CV and DCI [101, 102]. In vitro administration of CBD to rat cortical neuron cultures exposed to toxic levels of glutamate was found to reduce glutamate neurotoxicity by 60%, an efficacy which was significantly higher than the dietary antioxidants ascorbate (vitamin C) and α-tocopherol [103]. CBD attenuated brain excitotoxicity in a pig model of HI by reducing glutamate levels and preventing an increase in the glutamate/N-acetylaspartate ratio [72, 73]. In contrast, CBD did not attenuate the increased levels of glutamate in a mouse model of MCAO, yet it provided neuroprotective effects by enhancing cerebral microcirculation and inhibition of myeloperoxidase activity in neutrophils [92]. Further studies will be necessary to determine if CBD’s positive effects on various forms of HI injury are mediated by limiting glutamate release, activation of the glutamate receptors, or other mechanism(s). Nonetheless, the potential attenuation of excitotoxicity by CBD may provide therapeutic benefits in EBI pathology as well as later development of DCI.

### Reactive Oxygen Species

The generation of reactive oxygen species (ROS) is believed to play a significant role in the pathophysiology of SAH. The release of oxyhemoglobin acutely after aneurysm rupture causes auto-oxidation to produce oxygen (O_2_^−^) and hydrogen peroxide (H_2_O_2_), while also deriving ferrous catalyzed hydroxyl radicals that contribute to the increased concentration of intracellular calcium [84, 104, 105], stimulating CV and thrombus formation. Furthermore, ischemia can induce mitochondrial dysfunction and the release of free electrons capable of forming O_2_^−^ and H_2_O_2_ [106], toxic oxygen derivatives that induce oxidative stress. Breakdown (via superoxide dismutase) and binding (via iron chelators) of these reactive molecules have shown to be neuroprotective in SAH animal models by reducing oxidative stress, attenuating lipid peroxidation, and preventing CV [107–110]. An important, innate pathway for modulating the concentration of extracellular ROS is glutathione-peroxidase (GSH-Px)-catalyzed reactions, where glutathione (GSH) is used as a reducing agent for ROS. In experimental SAH models, GSH-Px activity is reduced in the cortex 48 h following SAH, and increasing GSH-Px activity reduced EBI, oxidative stress, and CV development [111–113].

CBD is known to be a potent antioxidant via its ability to reduce the generation of ROS. In multiple mouse models of disease, CBD reduces the expression of superoxide-generating enzymes, attenuates NADPH oxidase mRNA expression, decreases lipid peroxide proliferation, and reduces the generation of ROS [24, 67, 69]. In coronary artery endothelial cells, CBD attenuates high glucose-induced superoxide generation [66]. Although these models are not examining the brain, other studies have analyzed CBD’s effects on neurons and human brain endothelial cells. CBD exhibited a dose-dependent attenuation of *tert*-butyl hydroperoxide–induced oxidative damage in neuronal cell cultures similar to that occurring in SAH pathophysiology [103]. Beyond reducing the generation of ROS and the expression of ROS-generating enzymes, CBD also provides antioxidant effects by modulating the GSH-Px pathway. In both diabetic cardiomyopathy and hepatic I/R models, CBD restores the pathologic decrease in GSH [67, 79]. In newborn HI pig brains, Pazos et al. [72] showed CBD reduced oxidative stress by preventing an HI-induced decrease in the GSH/creatine ratio and an increase in protein carbonylation. Taken together, CBD has reproducibly been shown to reduce ROS levels, which may prove beneficial following SAH.

### Apoptosis

Death of brain cells, via apoptosis, necrosis, and autophagy, occurs early after SAH and significantly influences patient outcomes [114]. Caspases are a family of proteases that play important roles in programmed cell death and high serum concentrations may predict poor SAH patient outcomes and severity [115]. The presence of cleaved caspase-3, the catalytic activated form of the enzyme, is evident within 10 min in an animal model of SAH in both vascular and parenchymal cells, increasing significantly more within 24 h and peaking 2–3 days post-bleed [109, 114]. Inhibiting proteolytic cleavage activation of caspase-3 activity, as observed throughout the brain, reduces EBI, neuronal apoptosis, oxyhemoglobin-induced apoptosis, neurological deficits, and cerebral damage 24–48 h after SAH in animals [116–120]. This widespread apoptotic activity in the brain identifies a significant need for therapeutic intervention of apoptotic activity following SAH.

CBD has a demonstrated ability to reduce apoptotic activity during pathologic states, both in the brain and other anatomic locations. Abrantes De Lacerda Almeida et al. [121] evaluated CBD’s neuroprotective effects in a germinal matrix hemorrhage rodent model and found that intraperitoneal administration of CBD reduced astrocyte reactivity and the number of caspase-3 positive astrocytes. A germinal matrix hemorrhage is a type of neonatal intraventricular hemorrhage (located near the lateral ventricles) that is anatomically close to, but distinct from, hemorrhages in the subarachnoid space. When administered for 11 weeks in diabetic mice with cardiomyopathy, CBD attenuated enhanced caspase-3 cleavage, caspase-3/7 activity, PARP activity, and DNA fragmentation [67]. CBD administered to cisplatin-induced nephrotoxic mice also reduced apoptosis as measured by caspase-3/7 activity and DNA fragmentation [69]. In hepatic I/R mouse models, CBD significantly reduced DNA fragmentation and the expression of caspase-3, while increasing the expression of survivin protein, an inhibitor of caspase [65, 79]. Furthermore, CBD reduced caspase-9 expression in an in vitro model of HI, accompanied by reduced neuronal cell death [73].

## Discussion

Cannabidiol holds great potential for combatting several pathologies that occur following SAH. The multifaceted pharmacologic mechanisms of CBD involve antagonizing molecules that are important contributors to acute brain injury, CV with subsequent DCI development, chronic inflammation, and delayed neurologic deficits. We hypothesize the anti-inflammatory effects of CBD are likely to be the most therapeutically beneficial in the potential treatment of SAH, as inflammation is a confounding factor in multiple aspects of disease pathology.

**Fig. 1:**
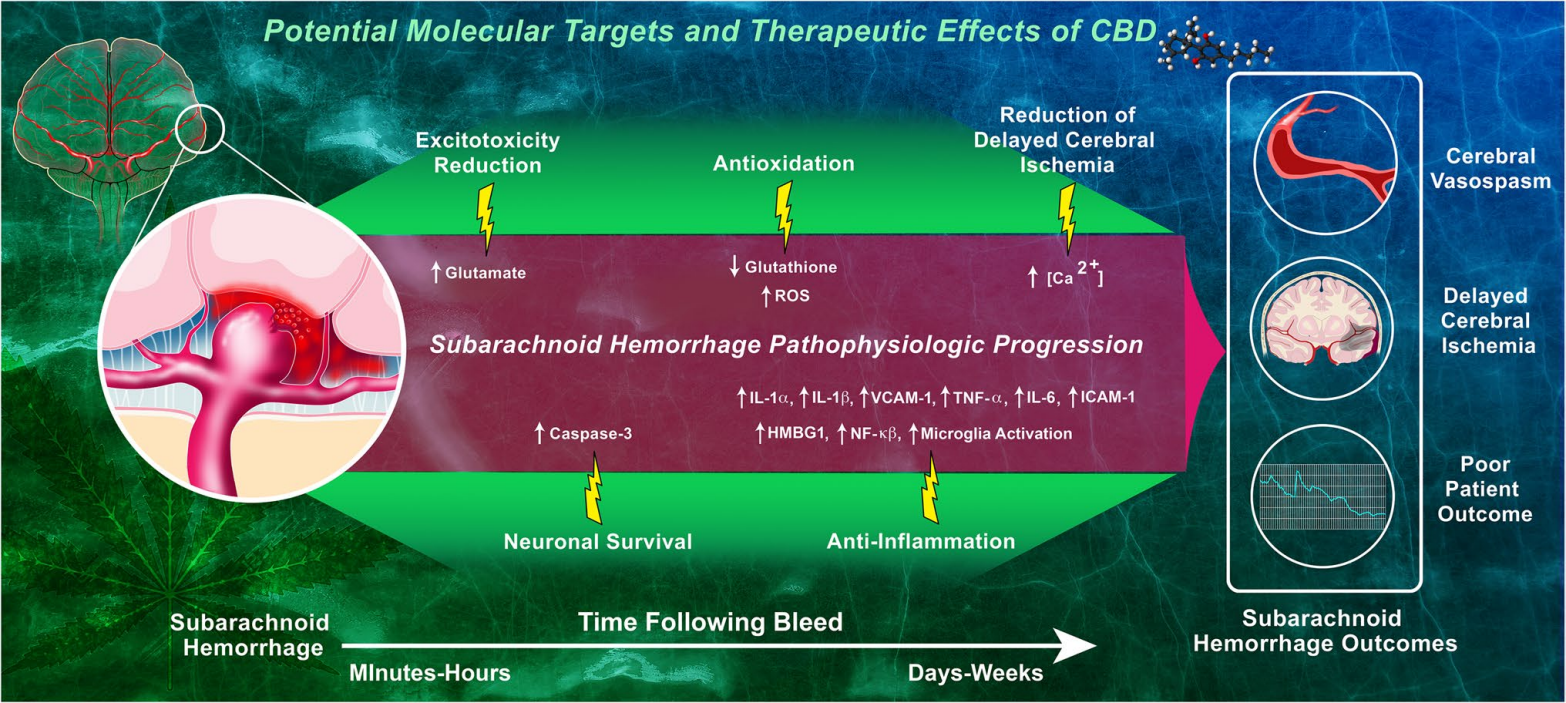
Potential molecular targets of CBD in SAH pathophysiology. Following a subarachnoid hemorrhage due to a ruptured aneurysm, a number of pathological changes occur (purple/red region), leading to cerebral vasospasm, delayed cerebral ischemia, and poor patient outcome. CBD’s potential effects to combat SAH (green region) are mediated by reversing or decreasing these pathologic changes (yellow bolts). A relative timeline of these pathologic changes is displayed at the bottom

CBD is a unique anti-inflammatory agent, particularly in the setting of SAH, because of its diverse mechanism profile that combats inflammation by both directly attenuating the immune response and indirectly protecting against events that would later stimulate inflammation (i.e., oxidative stress, apoptosis). Decreasing the expression of pro-inflammatory cytokines will help attenuate ongoing local inflammation as well as the synthesis of endothelial adhesion molecules and subsequent inflammatory cell infiltration into the brain parenchyma. These effects would be beneficial in preventing inflammation in the subacute phase of SAH since phagocyte infiltration is a part of the innate immune response that may contribute to early inflammation following SAH. In fact, recent studies have found appealing evidence of specific cytokines, most notably IL-1Rα, associated with poor patient outcomes following SAH, and large trials that target the IL-1 receptor are currently recruiting study participants [68, 122, 123]. The modulation of activated microglia, a known effect of CBD, may benefit both the acute phase by decreasing pro-inflammatory microglial activity and the subacute/chronic phases by inducing anti-inflammatory activity, which may help attenuate DCI and apoptosis. Distinctive from an innate response, these cells promote the migration of adaptive immune T cells and antigen-presenting dendritic cells, which stimulate the immune response even further, creating a cycle of chronic inflammation. Although efforts to combat SAH by reducing inflammation with other immunosuppressive agents have provided variable results [124], we believe that CBD’s anti-inflammatory properties along with its unique profile of other potentially therapeutic mechanisms may allow for more promising results. The specific molecular targets that produce these effects as well as those that produce CBD’s neuroprotective and vascular effects in SAH pathophysiology are depicted in Fig. 1.

As we have described, there is considerable evidence to suggest CBD also has neuroprotective effects against cell death, excitotoxicity, and oxidative stress. These pathologic mechanisms begin to occur as early as minutes following the initial bleed in SAH due to early leakage of oxyhemoglobin out of the vasculature with concomitant ischemic damage to neurons. Ischemic neurons subsequently die and release intracellular contents, including glutamate, that predispose cortical depolarization and environmental stress. Free oxyhemoglobin undergoes oxidative changes as previously described that leads to further exacerbated environmental stress via free radicals, predisposes vascular cells to undergoing future CV, and increases the risk of microthrombus formation. These mechanisms are potential contributors to adverse patient outcomes. Early intervention with CBD may therefore provide neuroprotective effects against ΕΒΙ in SAH and secondarily reduce DCI by combating these early pathologies.

Development of DCI, often as a result of CV, in the days following SAH puts the patient at a significantly increased risk of morbidity and mortality. Current treatments include the administration of the calcium channel blockers, nimodipine or verapamil. Nimodipine is the first line agent for patients presenting with vasospasm-induced ischemia, while verapamil is administered intra-arterially in the cerebral circulation for targeted treatment of DCI. CBD has been shown to regulate intracellular calcium concentrations, induce vasorelaxation in arteries, and protect BBB integrity via interactions with human brain microvasculature endothelial cells. Microvascular involvement, particularly of arterioles, occurs in SAH; however, the studies used in this review that evaluated microvascular effects of CBD did so in vitro and did not evaluate the in vivo effects of CBD in these vessels. Unfortunately, the current understanding of how CBD affects neurovascular tone in general is underdeveloped. Although both nimodipine and verapamil provide some benefits, they do not prevent DCI in all patients. As such, a therapeutic that acts through multiple mechanisms may prove more beneficial or act synergistically to better enhance the vasculature effects of these drugs via intracellular calcium modulation. There are no identified drug interactions between CBD and nimodipine or verapamil; however, CBD has been shown to affect the activity of various CYP450 enzymes including CYP3A4, the hepatic enzyme most responsible for the first-pass metabolism of calcium channel blockers [125]. Because of this, future studies should look to evaluate these potential interactions and, if pertinent, modify dosing regimens as needed. More so, CBD’s effects on CYP2C9 activity warrant administration considerations when co-administrating anti-epileptics for seizure prophylaxis and in patients who present on warfarin and need acute reversal. Fortunately, these effects should not impact the administration of heparin for DVT prophylaxis as it has unique metabolism pharmacology.

It is important to note that pre-morbid marijuana use identified by marijuana-positive urine drug screens was recently associated with stroke, DCI following aSAH, and possibly worse outcomes in patients with aSAH [126, 127]. These studies provide an alarming insight into the possible cerebrovascular risk of consuming cannabis; however, CBD is only one of > 100 cannabinoids in marijuana, all of which have uniquely variable pharmacologic effects. More so, the findings have a degree of confounding due to potential concomitant tobacco smoking, and the patients were all physiologically pre-conditioned with cannabis while having an abrupt halt in use following the insult. This sudden stop in exposure to the various components of cannabis could have then been what negatively influenced patient outcomes due to further alterations in physiologic homeostasis beyond what is induced by stroke, instead of the cannabis itself. It is therefore impossible to independently relate CBD to these findings, and future studies should look to identify whether purified CBD given in a clinical setting carries this association.

There are several limitations associated with integrating these findings into subclinical and clinical trials. We provided multiple mechanisms by which CBD may potentially provide therapeutic effects in SAH. However, we must address the plausibility that some of these effects, including early modulation of vascular tone and modulation of microglial activity including the phagocytosis of free oxyhemoglobin, may not be therapeutic or may even be harmful. Animal trials with variable administration strategies should be performed to identify whether these effects occur, and if so, how to avoid them based on administration modifications (i.e., delayed treatment to avoid early vasorelaxation). This review also summarizes pharmacologic findings that span multiple study modalities and diseases. It is challenging to extrapolate findings from animal or in vitro studies and successfully integrate them to produce similar findings in humans, especially with a pathology as intricate as SAH. This becomes even more challenging when different pathologies are being evaluated, as was done in the current review. As no studies were found to date that used CBD in SAH, the current comparison was warranted, and this limitation even further supports the need for future studies to evaluate CBD in SAH models.

The studies discussed here use a wide range of CBD doses and routes of administration. Online References 1–3 indicate the dose and route of administration of CBD as reported in the cited literature. Effects observed may be due, in part, to the concentration and method of administration of CBD, both in vivo and in vitro. Table 1 provides a summary of the in vivo studies referenced in this article that specifically evaluate CBD’s effects on the brain. These articles provide insight into the doses and routes of administration that have been typically used in previous in vivo CBD studies, which may be useful in future studies that aim to evaluate CBD’s in vivo effects in SAH and other CNS pathologies.

**Table 1:** All referenced studies demonstrating CBD’s in vivo effects in brain-localized pathologies

Article	Dosage	Model	Time/route of administration	Pathology being evaluated	CBD molecular/cellular interactions & physiologic effects
Hayakawa, 2008.^52^	0.1, 1, & 3 mg/kg	Male ddY mice	Before & 3 h after occlusion (*i.p.*)	Left MCA occlusion	↓HMBG1, ↓MPO, ↓microglia activity
Mecha, 2013.^53^	5 mg/kg	Female SJL/J mice	1–7 & 1–10 days post-infection (*i.p.*)	Demyelination	↓VCAM, ↓TNF-α, ↓IL-1β, ↓microglia activity
Martín-Moreno, 2011.^54^	20 mg/kg	C56/B16 mice	Daily for 1 week and 3 × /week for the next 2 weeks after Aβ injection	Alzheimer’s disease	↓IL-6 mRNA, ↓cognitive deficit
Pazos, 2013.^**72**^	1 mg/kg	Newborn pigs	30 min after HI (*i.v.*)	Hypoxia–ischemia	↓Glu/NAA ratio, ↓IL-1, prevented ↓GSH/creatine ratio
Lafuente, 2011.^78^	0.1 mg/kg	Newborn pigs	15 & 240 min after HI (*i.v.*)	Hypoxia–ischemia	↓TNF-α, ↓neuronal cell death
Hayakawa, 2009.^81^	3 mg/kg	Male ddY mice	Daily 1–14, 3–12, & 5–10 days after occlusion (*i.p.*)	Left MCA occlusion	↓HMBG1
Hayakawa, 2007.^92^	1 & 3 mg/kg	Male ddY mice	Right before, 3 & 4 h after occlusion (*i.p.*)	MCA occlusion	↑CBF, ↓MPO, no effect on excitotoxicity
Yokubaitis, 2021.^93^	0.3, 1, 3 mg/kg	C57B/6 mice	1 h before and 24 h after induction	Cold light ischemia	↓infarct size, ↓microglia activity
Abrantes De Lacerda Almeida, 2019.^121^	1 mg, 10 mg, 10 mg/kg	Wester rats	Multiple groups; pretreatment, 1 h after induction, daily × 7 days	Germinal Matrix Hemorrhage	↓astrocyte reactivity, ↓apoptotic cells, ↓caspase-3

As we suggest in this review, future investigations into the effects of CBD in experimental models of SAH are needed. There is a variety of animal models of SAH currently available [128], and ex vivo and in vitro studies are utilized to evaluate the effects of SAH, blood products, compounds, etc., on isolated arteries and various brain cell types. A combination of models will provide the best translatability of findings to clinical trials.

## Conclusion

While controversy exists around the use of CBD as a therapeutic, we hypothesize CBD’s anti-inflammatory, vascular, and neuroprotective effects are all plausible mechanisms by which post-SAH critically ill patients may benefit. We suggest further research on CBD administration be performed, specifically following SAH, to verify these findings and expand the knowledge of in vivo effects of CBD.

## Supplementary Information

Below is the link to the electronic supplementary material.

Supplementary file1 (PDF 247 KB)
