# Supplementary material for: Cannabidiol’s Multifactorial Mechanisms Has Therapeutic Potential for Aneurysmal Subarachnoid Hemorrhage: a Review
Source: Transl Stroke Res. 2022 Sep 15;14(3):283–96. doi: 10.1007/s12975-022-01080-x (PMC10160197; doi:10.1007/s12975-022-01080-x)
Supplement: Supplementary file 1 — Supplementary file1 (PDF 247 KB) [file 12975_2022_1080_MOESM1_ESM.pdf]

Cannabidiol's Multifactorial Mechanisms has Therapeutic Potential for Aneurysmal Subarachnoid Hemorrhage: A Review

Journal: Translational Stroke Research

Nicholas Henry[1], Justin F. Fraser[2-4,6,7], Joseph Chappell[8], Tamra Langley[5], and Jill M. Roberts[2-3,7*]

College of Medicine[1] Departments of Neurosurgery[2], Neuroscience[3], Radiology[4], Anesthesiology[5], Neurology[6], and Center for Advanced Translational Stroke Science[7], College of Pharmacy Department of Pharmaceutical Sciences[8], University of Kentucky, Lexington, KY, USA

*Corresponding Author Jill M. Roberts
Jill.roberts@uky.edu

Table OR1: Referenced Articles Demonstrating CBD's Anti-Inflammatory Effects							
	**Reference Article #**	**Model**	**CBD Molecular/Cellular Interactions and Physiologic effects**	**Anatomical Location/Cell Type Evaluated**	**Specific Pathology being Evaluated**	**ROA**	**Dosage**
**Microglia**							
	Hassan, 2014.[42]	Male C57BL Mice	↑TRPV1/2, ↑Microglia Phagocytosis	Isolated Brain and Spinal Cord BV-2 Cells	N/A	*in vitro*	10µM
	Juknat, 2013.[47]	Cultured Mice Cells	Many, See article*	BV-2 Cells Assay	*E. Coli* LPS induced inflammation	*in vitro*	10µM
	Kozela, 2010.[48]	Cultured Mice Cells	↓IL-1β/6, ↓IFN-β, ↓NF-κβ, ↑STAT3, ↓STAT1	BV-2 Cells Assay	*E. Coli* LPS induced inflammation	*in vitro*	10µM
	Liou, 2008.[49]	Newborn Rats	↓Adenosine uptake, ↓TNF-α,	Retinal Microglia Cells Assay; Retina	LPS induced inflammation	*in vitro*; i.p.	1µM; 1mg/kg
	Dos-Santos-Pereira, 2020.[51]	Cultured Mice Cells	↓ROS, ↓NF-κβ, ↓Microglia Activity	Microglia Cells Assay	LPS induced inflammation	*in vitro*	1-10µM
	Hayakawa, 2008.[52]	Male ddY Mice	↓Microglia activity, ↓HMBG1, ↓MPO activity	Brain	Left MCA Occlusion	i.p.	0.1, 1, 3mg/kg
	Mecha, 2013.[53]	Female SJL/J Mice	↓VCAM, ↓CCL2/5, ↓TNF-α, ↓IL-1β, ↓Microglia Activity	Brain and Spinal Cord	demyelinating disease	i.p.	5mg/kg
	Martín-Moreno, 2011.[54]	Cultured Mice Cells	Microglial Activity Modulation	Microglia Cells Assay	N/A	*In vitro*	100nM
		C56/B16 Mice	↓IL-6 mRNA, ↓cognitive deficit	Brain	Alzheimer's disease	i.p.	20mg/kg
	Carrier, 2006.[55]	Cultured Mice Cells	↓ Adenosine and ↓ Thymidine Uptake into Microglia	Bone Marrow Microglia and Macrophages	N/A	*in vitro*	0.1-3µM
		Male ICR or C57BL/6 Mice	↓ TNF-α	Serum	LPS induced inflammation	i.p.	1 mg/kg
	Juknat, 2012.[56]	Cultured Mice Cells	Many, See article*	BV-2 Cells Assay	N/A	*in vitro*	10µM
	Rimmerman, 2013.[57]	Cultured Mice Cells	↓ VDAC1, ↑BV-2 i[Ca], ↑BV-2 Cell Death	BV-2 Cells Assay	N/A	*in vitro*	5µM; 10µM

**Adhesion Molecules**							
	Mecha, 2013.[53]	Female SJL/J Mice	↓VCAM, ↓TNF-α, ↓IL-1β, ↓Microglia Activity	Brain & Spinal Cord	demyelinating disease	i.p.	5mg/kg
	Hind, 2016.[60]	Cultured Human Cells	↓ OGD-induced ↑ in BBB permeability	Brain Endothelium and Astrocytes	Oxygen-Glucose Deprivation	*in vitro*	10μM
	Mukhopadhyay, 2011.[65]	Male C57/BL6J Mice	↓NF-κβ, ↓TNF-α, ↓ICAM-1, ↓neutrophil infiltration, ↓DNA fragmentation	Liver	Ischemia/Reperfusion	i.p.	3, 10mg/kg
	Rajesh, 2007.[66]	Cultured Human Cells	↓NF-κβ, ↓monocyte migration	Human Coronary Artery Epithelial Cells	High Glucose Exposure	*in vitro*	4μM, 0-6μM
		Cultured Human Cells	↓superoxide, ↓VCAM-1, ↓ICAM-1	Human Coronary Artery Epithelial Cells	High Glucose Exposure	*in vitro*	4μM, 0-6μM
	Rajesh, 2010.[67]	Male C57/BL6J Mice	↓ICAM-1, ↓VCAM-1, ↓TNF-α, ↓ROS, ↓caspase 3,	Myocardial Tissue	Type 1 Diabetic Cardiomyopathy	i.p.	1,10, 20mg/kg
		Cultured Human Cells	↓superoxide generation, ↓NF-κβ, ↓apoptosis	Cardiomyocytes	High Glucose Exposure	*in vitro*	4μM
**Interleukins**							
	Juknat, 2013.[47]	Cultured Mice Cells	Many, See article*	BV-2 Cells Assay	*E. Coli* LPS induced inflammation	*in vitro*	10μM
	Kozela, 2010.[48]	Cultured Mice Cells	↓IL-1β/6, ↓IFN-β, ↓NF-κβ, ↑STAT3, ↓STAT1	BV-2 Cells Assay	*E. Coli* LPS induced inflammation	*in vitro*	10μM
	Mecha, 2013.[53]	Female SJL/J Mice	↓VCAM, ↓CCL2/5, ↓TNF-α, ↓IL-1β, ↓Microglia Activity	Brain and Spinal Cord	demyelinating disease	i.p.	5mg/kg
	Pan, 2009.[69]	C57/BL6J Mice	↓superoxide generation, ↓caspase 3, ↓DNA fragmentation, ↓TNF-α, ↓IL-1β	Serum and Kidney	Nephrotoxicity	i.p.	2.5-10mg/kg
	Jiang, 2021.[70]	Rat	↓neurologic deficit, ↓BBB disruption, ↓H20 requirement, ↓TNF-α, ↓IL-1β	Brain	TBI	i.p.	5, 10, or 20 mg/kg

	Sacerdote, 2005.[71]	Cultured Mice Cells	↑IL-12, ↓IL-10	Murine Peritoneal Macrophages	fMLP treatment	*in vitro*	0.005, 0.05, 0.5, 1, 5µM
		Swiss Male Mice	↑IL-12, ↓IL-10, ↓macrophage chemotaxis	Harvested Peritoneal Macrophages	fMLP treatment	p.o. or i.p.	15, 30mg/kg
	Pazos, 2013.[73]	Newborn Pigs	Prevented ↓ in GSH/Creatine ratio, ↓Glu/NAA ratio, ↓IL-1	Brain	Hypoxia-Ischemia	i.v.	1mg/kg
	Castillo, 2010.[73]	Newborn C57BL6 Mice	↓Glutamate, ↓caspase 9, ↓IL-6, ↓TNF-α	Brain	Hypoxia-Ischemia	*in vitro*	0.1-1000µM (primarily 100µM)
**TNF-α**							
	Malfait, 2000.[24]	C57/BL Mice	↓IFN-γ, ↓TNF-α, ↓lymphocyte proliferation, ↓ROS	Synovial Cells	Collagen-induced arthritis	o.p. & i.p.	5, 25mg/kg
	Liou, 2008.[49]	Newborn Rats	↓Adenosine uptake, ↓TNF-α,	Retinal Microglia Cells Assay; Retina	LPS induced inflammation	*in vitro*; i.p.	1µM; 1mg/kg
	Mecha, 2013.[53]	Female SJL/J Mice	↓VCAM, ↓CCL2/5, ↓TNF-α, ↓IL-1β, ↓Microglia Activity	Brain and Spinal Cord	demyelinating disease	i.p.	5mg/kg
	Carrier, 2006.[55]	Cultured Mice Cells	↓ Adenosine and ↓ Thymidine Uptake into Microglia	Bone Marrow Microglia and Macrophages	N/A	*in vitro*	0.1-3µM
		Male ICR or C57BL/6 Mice	↓ TNF-α	Serum	LPS induced inflammation	i.p.	1 mg/kg
	Mukhopadhyay, 2011.[65]	Male C57/BL6J Mice	↓NF-κβ, ↓TNF-α, ↓ICAM-1, ↓neutrophil infiltration, ↓DNA fragmentation	Liver	Ischemia/Reperfusion	i.p.	3, 10mg/kg
	Pan, 2009.[69]	C57/BL6J Mice	↓superoxide generation, ↓caspase 3, ↓DNA fragmentation, ↓TNF-α, ↓IL-1β	Serum and Kidney	Nephrotoxicity	i.p.	2.5-10mg/kg

	Jiang, 2021.[70]	Rat	↓neurologic deficit, ↓BBB disruption, ↓H20 requirement, ↓TNF-α, ↓IL-1β	Brain	TBI	i.p.	5, 10, or 20 mg/kg
	Castillo, 2010.[73]	Newborn C57BL6 Mice	↓Glutamate, ↓caspase 9, ↓IL-6, ↓TNF-α	Brain	Hypoxia-Ischemia	*in vitro*	0.1-1000μM (primarily 100μM)
	Lafuente, 2011.[78]	Newborn Pigs	↓TNF-α, ↓neuronal cell death	Brain	Hypoxia-Ischema	i.v.	0.1mg/kg
	Fouad, 2011.[79]	Male Sprague-Dawley Rats	↓ TNF-α, ↓lipid peroxidation, prevented ↓ in glutathione	Liver	ischemia/Reperfus ion	i.v.	5mg/kg
**HMBG1**							
	Hayakawa, 2008.[52]	Male ddY Mice	↓Microglia activity, ↓HMBG1, ↓MPO activity	Brain	Left MCA Occlusion	i.p.	0.1, 1*, & 3mg/kg
	Hayakawa, 2009.[81]	Male ddY Mice	↓HMBG1	Brain	Left MCA Occlusion	i.p.	3mg/kg
**Transcription Factors**							
	Juknat, 2013.[47]	Cultured Mice Cells	Many, See article*	BV-2 Cells Assay	*E. Coli* LPS induced inflammation	*in vitro*	10μM
	Kozela, 2010.[48]	Cultured Mice Cells	↓IL-1β/6, ↓IFN-β, ↓NF-κβ, ↑STAT3, ↓STAT1	BV-2 Cells Assay	*E. Coli* LPS induced inflammation	*in vitro*	10μM
	Dos-Santos-Pereira, 2020.[51]	Cultured Mice Cells	↓ROS, ↓NF-κβ, ↓Microglia Activity	Microglia Cells Assay	LPS induced inflammation	*in vitro*	1-10μM
	Mukhopadhya y, 2011.[65]	Male C57/BL6J Mice	↓NF-κβ, ↓TNF-α, ↓ICAM-1, ↓neutrophil infiltration, ↓DNA fragmentation	Liver	Ischemia/Reperfus ion	i.p.	3, 10mg/kg
	Rajesh, 2007.[66]	Cultured Human Cells	↓NF-κβ, ↓monocyte migration	Human Coronary Artery Epithelial Cells	High Glucose Exposure	*in vitro*	4μM, 0-6μM
		Cultured Human Cells	↓superoxide, ↓VCAM-1, ↓ICAM-1	Human Coronary Artery Epithelial Cells	High Glucose Exposure	*in vitro*	4μM, 0-6μM

	Rajesh, 2010.[67]	Male C57/BL6J Mice	↓ICAM-1, ↓VCAM-1, ↓TNF-α, ↓ROS, ↓caspase 3,	Myocardial Tissue	Type 1 Diabetic Cardiomyopathy	i.p.	1,10, 20mg/kg
		Cultured Human Cells	↓superoxide generation, ↓NF-κβ, ↓apoptosis	Cardiomyocytes	High Glucose Exposure	*in vitro*	4μM

Table OR2: Referenced Articles Demonstrating CBD's Vascular Effects							
	**Reference Article #**	**Model**	**CBD Molecular/Cellular Interactions and Physiologic effects**	**Anatomical Location/Cell Type Evaluated**	**Specific Pathology being Evaluated**	**ROA**	**Dosage**
**Hemodynamics and Ca2+**							
	Ryan, 2009.[87]	Lister-Hooded Rats	Bidirectional i[Ca2+] modulation	Hippocampal Cells	N/A	*in vitro*	0.1, 1µM
	Ryan, 2009.[87]	Lister-Hooded Rats	prevent [Ca2+] oscillations in high excitable state	Hippocampal Cells	N/A	*in vitro*	0.1, 1µM
	Al Suleimani, 2015.[88]	Male Wistar Rats	Vasorelaxation via Ca^{2+}/K^{+} Channels	SMA Endothelial Cells	Methoxamine-induced vasoconstriction	*ex vivo*	0.001-100µM
	MacIntyre, 2014.[89]	Male Fischer 344 Rats	Vasorelaxation via Ca^{2+}/K^{+} Channels	Retinal Micro-vasculature	Endothelin 1-induced vasoconstriction	*ex vivo*	10mM
	Wheal, 2014.[90]	ZDF Rats	Vasorelaxation via COX1/2 and EP4 receptor mechanisms	Femoral Artery	Diabetes	*ex vivo*	10µM
**Ischemia**							
	Hayakawa, 2008.[52]	Male ddY Mice	↓Microglia activity, ↓HMBG1, ↓MPO activity	Brain	Left MCA Occlusion	i.p.	0.1, 1, 3mg/kg
	Hayakawa, 2009.[81]	Male ddY Mice	↓HMBG1, ↓ischemic damage	Brain	Left MCA Occlusion	i.p.	3mg/kg
	Hayakawa, 2007.[92]	Male ddy Mice	↑CBF, ↓MPO activity, no effect on glutamate excitotoxicity	Brain	MCA Occlusion	i.p.	1, 3mg/kg
	Yokubaitis, 2021.[93]	C57B/6 Mice	↓infarct size, ↓Microglia activity	Brain	Cold light ischemia	i.p.	0.3, 1, 3mg/kg
	Feng, 2015.[94]	Rabbits	↓lymphocyte infiltration, ↓apoptosis, ↑blood flow, ↑perfusion density	Myocardium	Ischemia/ reperfusion	i.v.	100µg/kg
	Walsh, 2010.[95]	Male Sprague-Dawlet Rats	↓arrythmias, ↓infarct size, ↓platelet aggregation	Myocardium	Coronary Artery Occlusion	i.v.	10 or 50µg/kg

	Gonca, 2015.[96]	Male Wistar Rats	↓tachycardia, ↓arrythmia length, mediated via A1 receptor	Myocardium	Ischemia/ reperfusion	i.v.	50µg/kg

Table OR3: Referenced Articles Demonstrating CBD's Neuroprotective Effects

	Reference Article #	Model	CBD Molecular/Cellular Interactions and Physiologic effects	Anatomical Location/Cell Type Evaluated	Specific Pathology being Evaluated	ROA	Dosage
**Excito-toxicity**							
	Pazos, 2013.[72]	Newborn Pigs	Prevented ↓ in GSH/Creatine ratio, ↓Glu/NAA ratio, ↓IL-1	Brain	Hypoxia-Ischemia	i.v.	1mg/kg
	Castillo, 2010.[73]	Newborn C57BL6 Mice	↓Glutamate, ↓caspase 9, ↓IL-6, ↓TNF-α	Brain	Hypoxia-Ischemia	*in vitro*	0.1-1000µM
	Hayakawa, 2007.[92]	Male ddy Mice	↑CBF, ↓MPO activity, no effect on glutamate excitotoxicity	Brain	MCA Occlusion	i.p.	1, 3mg/kg
	Hampson, 1998.[103]	Newborn Wistar Rats	↓excitotoxicity, ↓H2O2 oxidative damage	Cortical Neuron Cultures	Glutamate-induced excitotoxicity	*in vitro*	10µM
**Reactive Oxygen Species**							
	Malfait, 2000.[24]	C57/BL Mice	↓IFN-γ, ↓TNF-α, ↓lymphocyte proliferation, ↓ROS	Synovial Cells	Collagen-induced arthritis	o.p. & i.p.	5, 25mg/kg
	Rajesh, 2007.[66]	Cultured Human Cells	↓NF-κβ, ↓monocyte migration	Human Coronary Artery Epithelial Cells	High Glucose Exposure	*in vitro*	4µM, 0-6µM
		Cultured Human Cells	↓superoxide, ↓VCAM-1, ↓ICAM-1	Human Coronary Artery Epithelial Cells	High Glucose Exposure	*in vitro*	4µM, 0-6µM
	Rajesh, 2010.[67]	Male C57/BL6J Mice	↓ICAM-1, ↓VCAM-1, ↓TNF-α, ↓ROS, ↓caspase 3,	Myocardial Tissue	Type 1 Diabetic Cardiomyopathy	i.p.	1,10, 20mg/kg
		Cultured Human Cells	↓superoxide generation, ↓NF-κβ, ↓apoptosis	Cardiomyocytes	High Glucose Exposure	*in vitro*	4µM
	Pan, 2009.[69]	C57/BL6J Mice	↓superoxide generation, ↓caspase 3, ↓DNA	Serum and Kidney	Nephrotoxicity	i.p.	2.5-10mg/kg

			fragmentation, ↓TNF-α, ↓IL-1β				
	Pazos, 2013.[72]	Newborn Pigs	Prevented ↓ in GSH/Creatine ratio, ↓Glu/NAA ratio, ↓IL-1	Brain	Hypoxia-Ischemia	i.v.	1mg/kg
	Fouad, 2011.[79]	Male Sprague-Dawley Rats	↓ TNF-α, ↓lipid peroxidation, prevented ↓ in glutathione	Liver	ischemia/Reperfusio n	i.v.	5mg/kg
	Hampson, 1998.[103]	Newborn Wistar Rats	↓excitotoxicity, ↓H2O2 oxidative damage	Cortical Neuron Cultures	Glutamate-induced excitotoxicity	*in vitro*	10µM
**Cell Death**							
	Mukhopadhyay, 2011.[65]	Male C57/BL6J Mice	↓NF-κβ, ↓TNF-α, ↓ICAM-1, ↓neutrophil infiltration, ↓DNA fragmentation	Liver	Ischemia/Reperfusio n	i.p.	3, 10mg/kg
	Rajesh, 2010.[67]	Male C57/BL6J Mice	↓ICAM-1, ↓VCAM-1, ↓TNF-α, ↓ROS, ↓caspase-3,	Myocardial Tissue	Type 1 Diabetic Cardiomyopathy	i.p.	1,10, 20mg/kg
		Cultured Human Cells	↓superoxide generation, ↓NF-κβ, ↓apoptosis	Cardiomyocytes	High Glucose Exposure	*in vitro*	4µM
	Pan, 2009.[69]	C57/BL6J Mice	↓superoxide generation, ↓caspase-3, ↓DNA fragmentation, ↓TNF-α, ↓IL-1β	Serum and Kidney	Nephrotoxicity	i.p.	2.5-10mg/kg
	Castillo, 2010.[73]	Newborn C57BL6 Mice	↓Glutamate, ↓caspase-9, ↓IL-6, ↓TNF-α	Brain	Hypoxia-Ischemia	*in vitro*	0.1-1000µM
	Fouad, 2011.[79]	Male Sprague-Dawley Rats	↓ TNF-α, ↓lipid peroxidation, prevented ↓ in glutathione	Liver	ischemia/Reperfusio n	i.v.	5mg/kg
	Abrantes De Lacerda Almeida, 2019.[121]	Wester Rats	↓astrocyte reactivity, ↓apoptotic cells, ↓Caspase-3	Brain	Germinal Matrix Hemorrhage	i.p.	1mg, 10mg, 10mg/kg
